# A diversified role for γδT cells in vector-borne diseases

**DOI:** 10.3389/fimmu.2022.965503

**Published:** 2022-08-16

**Authors:** Chen Chen, Aibao Chen, Yanan Yang

**Affiliations:** ^1^ Department of Microbiology, School of Basic Medical Sciences, Anhui Medical University, Hefei, China; ^2^ Department of Cell Biology, School of Life Sciences, Anhui Medical University, Hefei, China; ^3^ Department of Immunology, School of Basic Medical Sciences, Anhui Medical University, Hefei, China

**Keywords:** γδT cells, vector-borne diseases (VBDs), host immune response, infection, pathogens

## Abstract

Vector-borne diseases have high morbidity and mortality and are major health threats worldwide. γδT cells represent a small but essential subpopulation of T cells. They reside in most human tissues and exert important functions in both natural and adaptive immune responses. Emerging evidence have shown that the activation and expansion of γδT cells invoked by pathogens play a diversified role in the regulation of host-pathogen interactions and disease progression. A better understanding of such a role for γδT cells may contribute significantly to developing novel preventative and therapeutic strategies. Herein, we summarize recent exciting findings in the field, with a focus on the role of γδT cells in the infection of vector-borne pathogens.

## Introduction

**Figure 1:**
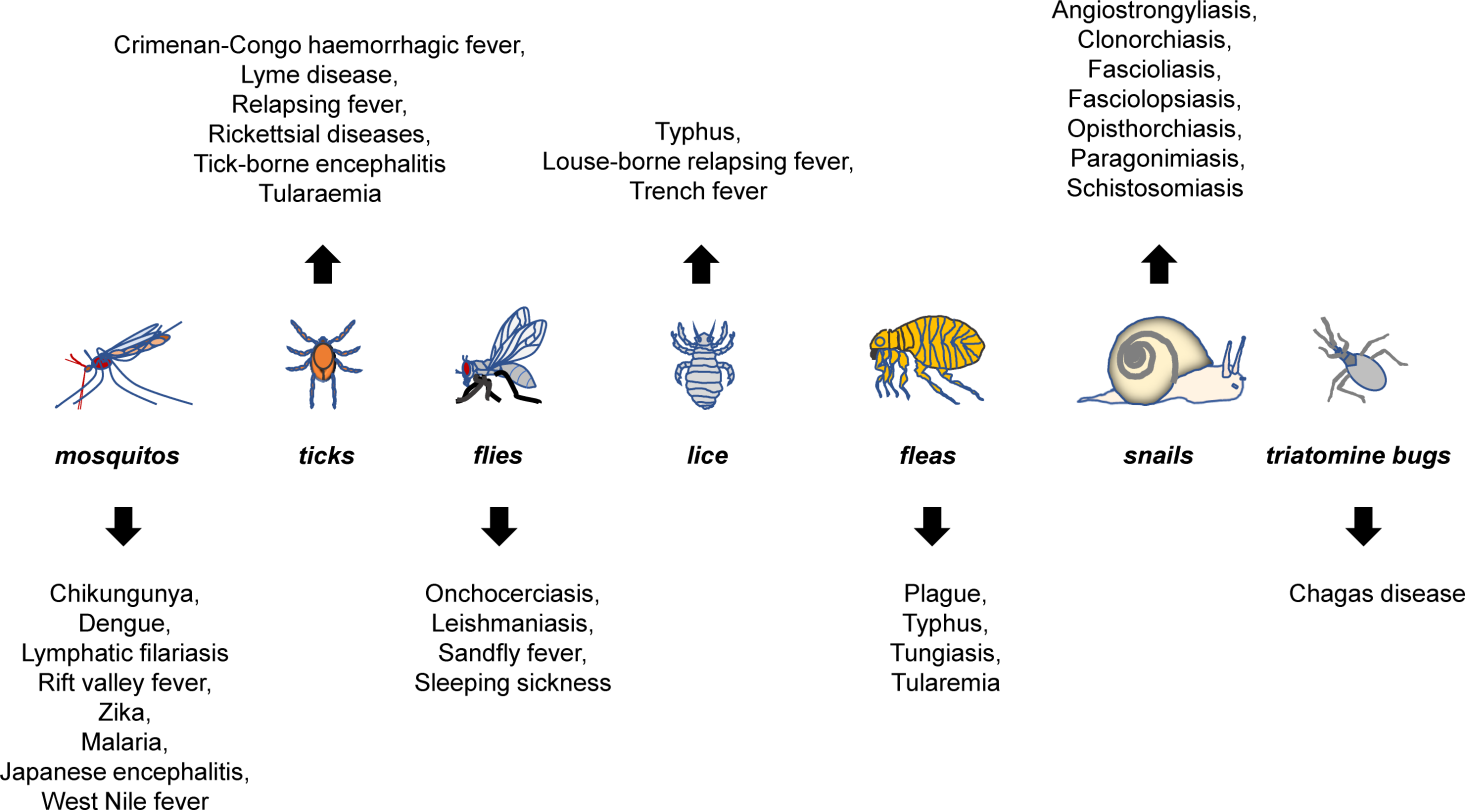
Major vector-borne diseases and their vectors. Mosquitos, ticks, fleas, flies, lice, snails, and triatomine bugs are best-characterized vectors that can carry pathogens for a variety of diseases. The listed are representative rather than a complete list of major vector-borne diseases that are known to be transmitted by each of the vectors.

Vector-borne emerging and re-emerging infectious diseases are major public health problems worldwide, accounting for more than one sixth of all infectious diseases (1, 2). They are caused by pathogens carried and transmitted by vectors, such as mosquitos, ticks, fleas, flies, lice, snails, and triatomine bugs (**Figure 1**).

WHO recently announced the spread of the vector-borne pathogens, primarily including parasites, viruses and bacteria (https://www.who.int/zh/news-room/fact-sheets/detail/vector-borne-diseases). Specifically, the parasites included were lymphatic filariasis (mosquito), schistosoma (aquatic snail), onchocodium filariasis (black fly) and trypanosoma (triatomine bug, tsetse fly) and etc; viruses include mosquito-borne chikungunya fever, dengue, lymphatic filariasis, Rift Valley fever, yellow fever, Zika, and tick-borne Crimean-Congo hemorrhagic fever virus, borrelia burgdorferi, tick-borne encephalitis virus, and etc.; and bacteria mainly include Typhoid, Coxella bainiensis, spot fever rickettsia, and etc. (**Figure 1**).

Vector-borne diseases may not be directly disseminated between humans. Under certain circumstances, they can be transmitted to different hosts through the bite by pathogen-infected vectors (3, 4). As emerging infectious diseases (including vector-borne diseases) have certain relationships with social and economic development (5), a better understanding of the emerging and recurrent infectious diseases, especially how these diseases are transmitted, has profound significance for both human health and social development.

In response to pathogen invasion, human immune system acts as an advanced structural and functional architecture, in which all components (e.g. immune organs and cells, inflammatory factors, humoral factors, cytokines and chemokines) are highly orchestrated towards eliminating the invaded pathogen (6). During the invasion, both innate and adaptive immune responses can be triggered. Compared to innate immunity, adaptive immunity utilizes antigen and antibody specificity to eliminate the pathogen, thereby maintaining a steady state of the host and creating immunological memory for combating potential re-invasion of the same pathogen.

Different leukocytes, for example B and T cells, are known to play differential roles during these processes (7). T cells can be categorized to conventional T cells (αβT cells) and unconventional T cells (γδT cells) according to the types of their cell surface antigen receptors (8). Although αβT and γδT originate (differentiate) from the same thymic precursors, there are huge differences of biological functions and structures between the two types of T cells. The αβT cell receptors are expressed by ~95% of the T cells in the spleen, lymph nodes, and circulation system, and by ~60-70% of T cells in the peripheral blood. They have α and β chains and exhibit MHC restriction during the recognition of antigens (9).

On the contrary, the γδT cells express γ and δ chains of T cell receptors, account for ~5-15% T cells in the peripheral blood, and do not have MHC restriction during the recognition of antigens (10, 11). At the initial stage during the invasion of pathogens, γδT cells apparently exert innate immunity functions (6), so that they can rapidly respond by recognizing some common antigen components expressed by the invading pathogens, including glycolipids, glycoproteins, and mycobacterial derivatives (12). Although γδT cells have been known to act primarily in innate immunity, more and more findings have shown that these cells also exert fundamental functions in adaptive immune responses, for instance, by secreting cytokines and presenting antigens. Therefore, they have been considered as a bridge connecting innate immunity and adaptive immunity. However, the biological functions of γδT cells are not entirely dependent on HLA recognition mechanism.

γδT accounts for only a small part of the T cell population and is widely distributed in different parts of the human body, such as skin and intestinal tract (7). Human γδT cells are mainly categorized by the usage of δ chain, whereas mouse γδT cells are often categorized by the usage of γ chains. As such, human γδT cells can be divided into γδ1, γδ2, and γδ3 T cells (13), with their distribution and function varying from each other (14). γδ1 T cells are mainly distributed in the mucosal epithelium and play an important role in cell infection by listeria and cytomegalovirus. γδ2 T cells are relatively high in peripheral blood γδT cells and show strong immune response to mycobacterium and influenza virus (15, 16). They destroy pathogens or infected cells by interacting rapidly with them (17, 18). γδ3 T cells, which account for a small proportion of γδT cell and are abundant in the liver, act during chronic viral infection (19).

The common and more harmful vector-borne diseases include dengue virus, Japanese encephalitis, Lyme disease and malaria. γδT cells play a key role in the host immune responses to the invasion of arbo-borne pathogens. More and more studies have shown that γδT cells are critical for antiviral and immunomodulatory activities in the first stage of arbo-borne pathogen infection. They are activated and participate in innate immune responses by producing cytokines associated with appropriate T-assisted responses during the early stages of microbial infection, either intracellular or extracellular (20). In addition to directly fighting against invading pathogens, γδT cells can also respond by recruiting other natural immune cells such as neutrophils and macrophages (21).

Infectious diseases are caused mainly by pathogenic microorganisms such as bacteria, viruses and parasites. γδT cells play important roles in responding to the invasion of common pathogens. Zhao and colleagues have summarized the role played by γδT cells in host responses to mycobacterium tuberculosis, Listeria monocytogenes, influenza viruses, HIV, EBV, and HBV (13). However, little is known about the effects of vectors on host γδT cells. Emerging vector-borne infectious diseases are an important part of emerging infectious diseases and have been in an intensified form globally. Many social and natural factors, including environmental pollution and modern transportation and logistics, make it more convenient for vectors to transmit arboreal pathogens.

Traditionally, many vector-borne diseases can be treated by antibiotics-based therapeutics. However, at least partly due to the antibiotics abuse in clinic, a variety of pathogens have developed resistance to common antibiotics, leading to poor clinical outcomes when using antibiotics to treat infected patients (22). To address this problem, it is critical for developing novel therapeutic approaches. In the past decades, scientists and clinicians have focused on the roles of conventional T cells-mediated immune responses during the pathogen infection. Notably, more and more evidence has uncovered previously-unrecognized key roles of unconventional T cells in this process. Therefore, we feel it is important to summarize recent progresses in the field investigating the functions of T cells (**Table 1**), especially the unconventional γδT cells, during the host immune responses to vector-borne pathogens, such as plasmodium, borrelia burgdorferi, and dengue fever, with a hope to accelerate our efforts in developing novel and effective clinical therapeutic strategies.

**Table 1:** Potential roles for **γδT cells** in vector-borne diseases.

Disease	Pathogen	Involved γδT cells and their potential roles	References
Chikungunya (mosquito-borne)	chikungunya fever virus	γδT cells; likely involved in promoting protective immunity	(23–25)
Rift Valley Fever (mosquito-borne)	Rift Valley Fever virus	CD11b^+^ γδT; may be critical for the host responses in sheep	(26–28)
Yellow fever (mosquito-borne)	Yellow fever virus	γδ2T cells; can respond quickly to virus infection and produce IFN-γ	(29)
Dengue fever (mosquito-borne)	Dengue fever virus	γδ2-T-cells; may serve as the early source of IFN-γ during dengue virus infection and promote the host immune responses by eliminating the virus-infected cells	(30–37)
Zika fever (mosquito-borne)	Zika virus	γδ2T; unclear	(38–40)
West Nile fever (mosquito-borne)	West Nile virus	γδT cells; may serve as the main source of IFN-γ and may also promote DC maturation and CD4^+^ T cell infiltration	(41–45)
Malaria (mosquito-borne)	plasmodium parasite	γδT cells, Vg9Vd2 subpopulation, and γδ2^+^ γδT cells; play both anti-pathogen and pathogenic roles	(46–60)
Lyme disease (tick-borne)	borrelia burgdorferi	γδT cells; may act indirectly through the actions of Toll-like receptors of DCs or monocytes, and may also act to activate the host acquired immunity during the infection of the pathogen	(61–66)
Tularaemia (tick-borne)	Francisella tularensis	γδT cells can be increased and maintained for up tyo a year in the peripheral blood from tularaemia patients	(67–69)
Leishmaniasis	leishmania	γδT cells; a potential role for γδT cells in eliminating the infected parasites, but long-term parasite infection may lead to γδT lymphoma	(70–73)
South American trypanosomiasis	Trypanosoma cruzi	γδT cells; may act by secreting IL-10 to facilitate host responses	(74)

## Immune responses of the host γδT cells to mosquito-borne pathogens

### Mosquito-borne viruses

#### Chikungunya

Chikungunya is caused by the infection of chikungunya fever virus. Its clinical manifestations include headache, fever, and serious joint pains (23–25). Vectors for chikungunya fever mainly include Aedes Aegypti and Aedes albopictus (also called Asian tiger mosquito) (75). Currently, there is no effective drugs or vaccines available for treating or preventing chikungunya (76). Different T cell family members play differential roles after the invasion of chikungunya fever. Rapidly accumulated CD8^+^ T cells have been detected in the joints of mice that are acutely infected by the virus to promote protective immunity, but the lack of CD8^+^ T seems to have no effect in preventing arthrophlogosis of the infected mice (77). On the contrary, the virus will not be able to induce joint diseases after the exhaustion of CD4^+^ T cells (77, 78). Activated CD4^+^ T cells have been shown to be implicated in the pathogenesis of arthrosis swell (77–79).

Unconventional γδT cells are also likely involved in promoting protective immunity in the host against chikungunya invasion. The numbers of γδT cells in the feet and lymph nodes are significantly increased after the mice are infected by chikungunya. Mice defective in γδT cells are more susceptible to chikungunya infection, exhibiting more severe foot swell and inflammation in the ankles, as well as increased oxidative damages, suggesting that γδT cells play critical roles in the protective immunity during the infection of chikungunya and subsequent inflammation and tissue damage (80).

#### Rift Valley Fever

Rift Valley Fever is a type of zoonosis caused by Rift Valley Fever virus, transmitted mainly by aedes and culex (26). Although most of infected patients only have minor fever, headache, and muscle pains, some patients may develop serious symptoms, including retinopathy, meningoencephalitis symptoms, and hemorrhagic fever (27). A possible role for γδT cells in Rift Valley Fever has been reported for infected sheep. Similar to other ruminants, the sheep’s γδT cells account for a major population of its peripheral blood mononuclear cells. When recombinant Rift Valley Fever vaccine has been injected into the sheep, the percentage of CD11b^+^ γδT in its peripheral blood mononuclear cells can be significantly increased, suggesting that these cells may be critical for the host responses in respond to the virus infection (28).

#### Yellow fever virus

Yellow fever virus belongs to the Flaviviridae, transmitted primarily by aedes and haemophilus mosquito. The symptoms for yellow fever commonly include fever, headache, jaundice, muscle pains, and emesia. Some patients may develop more serious symptoms and die quickly (29). So far, the YF-17D vaccine for yellow fever is probably one of the most effective vaccines. When it is inoculated into human hosts, γδ2T cells can respond quickly and produce IFN-γ within a week (28).

#### Dengue fever

Dengue virus is a mosquito-borne pathogen that is transmitted between hosts by mosquito bite (30). In clinic, most patients with slight infection will not have complications, and only a small population of patients will progress into severe disease states, exhibiting thrombocytopenia, end-organ damage, and other symptoms (31). critically ill patients commonly develop secondary infection, which is closely related with innate immunity (32, 33). In the host immunity, T cells are critical for eliminating pathogen invasion. In *in vitro* experiments, CD8^+^ αβT cells can respond to dengue virus, and many evidence have shown that these cells play important roles in the host responses (34, 35).

We know relatively less about the roles for γδ T cells in the dengue virus infection. Eleonora Cimini and colleagues analyzed peripheral blood mononuclear cells from 15 dengue fever patients, the results show a significant decrease of γδ2-T-cell frequency and an increase of failure markers. In addition, the ability of γδ2-T-cells to produce IFN-γ in response to the phosphor-antigen was limited (36). Interestingly, primary human γδT has been shown to be able to kill dengue virus *in vitro*, suggesting a potential role for these cells in the anti-dengue virus process. Further investigations by Chen-Yu Tsai and colleagues have shown that primary γδT cells serve as the early source of IFN-γ during dengue virus infection and promote the host immune responses by eliminating the virus-infected cells. Monocytes can act as helper cells to participate in the virus infection and enhance the immune reponses in an IL-18-dependent manner (37).

#### Zika virus

Similar to dengue virus, Zica virus is primarily transmitted by infected aedes mosquitoes in tropical and subtropical regions. The infection of Zica virus can cause Guillain-Barre syndrome, neuropathy and myelitis. The infection during pregnancy may lead to the development of microcephaly and other congenital abnormalities in fetuses and newborns (38), and there has been a lack of clear treatment strategy. Previous reports have shown significantly increased Th1, Th2, Th9, and Th17 during acute Zica infection (39), suggesting that conventional T cells may dominate the host responses to Zica invasion. However, it should be noted that Eleonora Cimini and colleagues have also specifically detected γδ2TCR in Zika virus-infected patients and a significantly increased expression level of CD3^+^CD4^−^CD8^−^ T cell subsets (40), implying a possible role for unconventional γδT cells.

#### West Nile virus

West Nile virus is a type of flavivirus, primarily transmitted by culex pipiens. Most West Nile virus-infected patients exhibit no significant symptoms (asymptomatic). However, the incidence of severe cases increases significantly in immunocompromised populations (41), and there is no targeted vaccine for such cases. γδT cells are thought to play an essential role in the early control of infection. They respond rapidly by producing large amounts of IFN-γ (42). In addition to serving as the main source of IFN-γ, γδT cells may also promote DC maturation and CD4^+^ T cell infiltration, as suggested by the observations that the expression of the dendric CD40, CD80, CD86 and MHC II molecules, as well as the expression of IL-12, are lower in γδT-deficient mice compared to those in wild-type mice. Furthermore, West Nile virus-induced activated γδT cells can promote the maturation of DC and the initiation and excitation of CD4^+^ cells (43) to combat against virus invasion.

Besides of above-mentioned roles, West Nile virus-activated γδT cells are also critical for limiting the invasion of the virus into the brain central vervous system, which is essential for protecting most infected-hosts from developing fatal encephalitis. Thomas Welte and colleagues have shown that, compared with young mice, older/aged mice are more susceptible to virus infection and have slower Vg1^+^ responses but more Vg4^+^ cells, which in turn produce TNF-α, a factor implicated in the destruction of the blood-brain barrier. On the other hand, low Vg4^+^ cells will allow the virus load in the brain, whereby reducing the mortality rate of virus-caused severe encephalitis (44). In the acquired immunity against West Nile virus infection, γδT cells also actively participate in the host defense process, and decreased memory responses of CD81^+^ T cells likely easily lead to secondary infection of the virus in γδT-deficient mice (45).

### Mosquito-borne parasite

#### Malaria

Malaria is estimated to affect more than 200 million people each year (46). It is an arbo-borne disease transmitted by the bite of mosquito-borne plasmodium parasite. Despite more and more significant progresses in the control and reduction of malaria cases in the past decade, it still remains a major threat to global health (47). After invading into the host, malaria parasites parasitize their spores in the liver of the host, then start to grow and eventually form merozoites to invade red blood cells, leading to significant clinical symptoms and death (48, 49).

**Figure 2:**
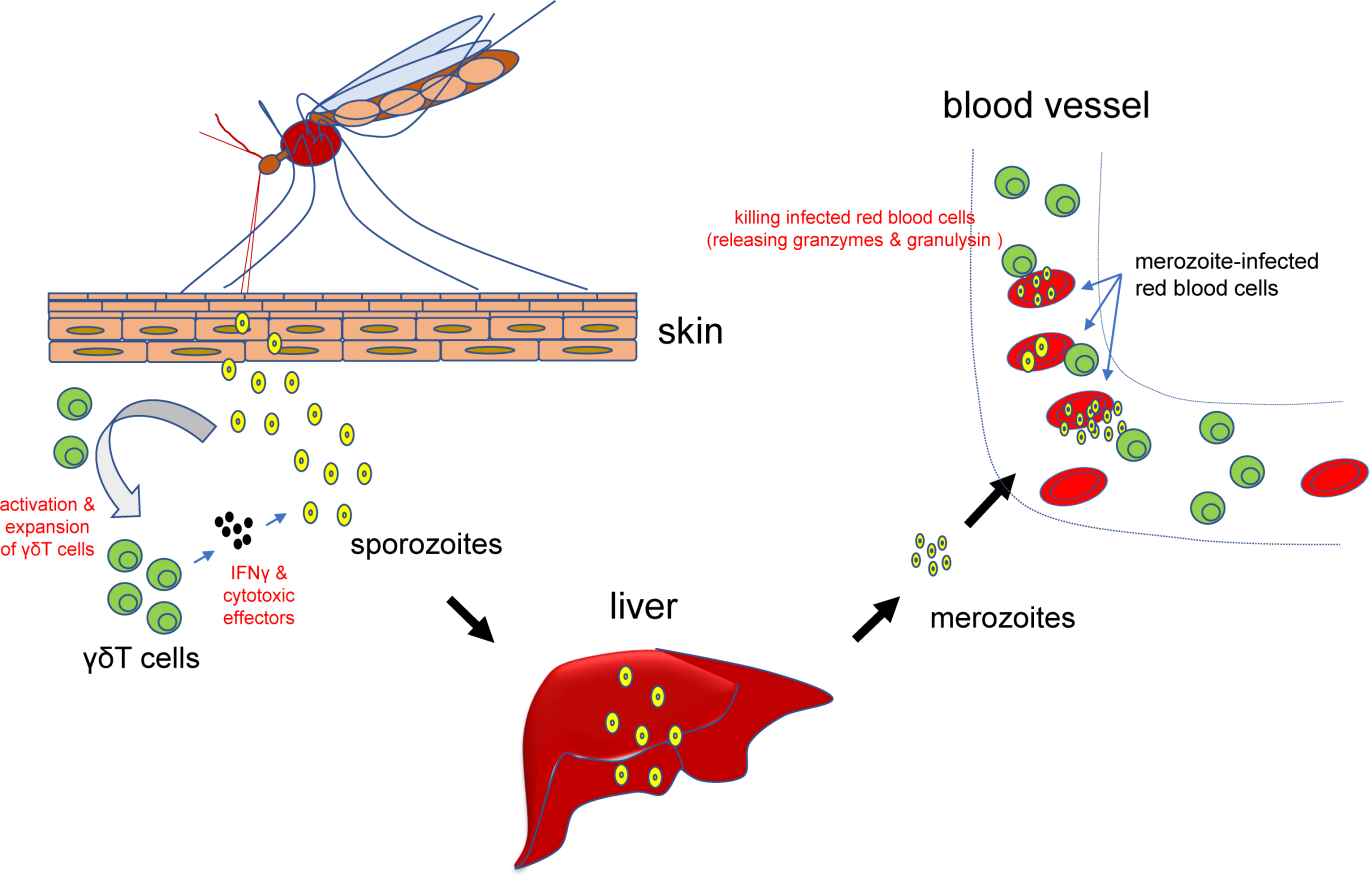
Roles for γδT cells in malaria parasites infection. At the early stage of infection, γδT cells can be activated and expanded and subsequently secret IFN-γ and other cytotoxic effectors to prevent or attenuate the infection. After the parasites have infected blood cells, activated γδT cells can also bind to the infected cells, release granzymes and granulysin, and kill the invaded plasmodium parasites and infected red blood cells.

The invasion of plasmodium parasites can cause complicated immune responses, including humoral and cellular immunity responses. We have very limited knowledge about the nature of these responses, especially the cellular immunity responses. Previously, it has been reported that γδT cells can be activated by phosphor-antigen of the parasites (50), leading to a quick increase of γδT cells, especially the Vg9Vd2 subpopulation (51, 52) (**Figure 2**). Such activation and/or expansion of γδT cells appear to be persistent after the invasion of the plasmodium parasites and can occur during secondary infections (53–55). It is already known that the main cause of the high morbidity and mortality of malaria patients is the successful survival and exponential proliferation of plasmodium parasites within the host blood. In the supernatants from the co-culture of plasmodium parasites, γδT cells can be specifically expanded and promoted to acquire the parasite-lysing potential through the up-regulated expression of IFN-γ and other cytotoxic effector proteins.

Subsequently, γδT cells can directly kill plasmodium parasite-infected red blood cells to prevent or attenuate further infections (56) (**Figure 2**). These killer cells can bind to the infected red blood cells, release granzymes and granulysin to kill the invaded plasmodium parasites (46). Therefore, a decrease of the numbers of γδT cells may reversely facilitate the tolerance of plasmodium parasites. Accordingly, repeated plasmodium parasites infection may contribute to the development of clinical immunity in children living in plasmodium parasites-infested regions, which is characterized by decreased numbers of patients with clear symptoms, accompanied by increased numbers of asymptomatic patients (57, 58).

During the repeated plasmodium parasites infections, the numbers of γδ2^+^ γδT cells will be decreased in the peripheral blood, along with down-regulated production of cytokines and up-regulated immune-related factors. As such, repeated plasmodium parasites infections in the childhood will drive a progressive loss of the γδ2^+^ γδT cells, leading to increased immune tolerance of the patients to plasmodium parasites (59).

Notably, besides above-mentioned anti-pathogen roles, γδT cells may also have a paradoxical role in driving or participating in the pathogenesis of cerebral malaria, as the incidences of cerebral malaria complications is lower in infants with lower γδT reactivity. Julie Ribot and colleagues have shown that the γδT-deficient mice are more resistant to the development of cerebral malaria when infected with the plasmodium berghei ANKA sporozoa. Conversely, the presence of γδT cells can enhance the production of the plasmodium immune factors at the stage of liver infection and subsequently promote the inflammation reactions at the blood infection stage (60). Together, these findings demonstrate that γδT cells can promote the pathogenesis of IFN-γ-dependent plasmodium infection.

### Immune responses of the host γδT cells to tick-borne pathogens

#### Borrelia burgdorferi

Compared to mosquito-borne pathogens, there have been less reports regarding the roles of γδT cells in the infection of tick-borne pathogens. The Lyme disease is the most frequently seen natural epidemic disease in the United States of America (61) and is transmitted through bites from different hosts by borrelia burgdorferi-carrying ticks (62). Initial symptoms after the pathogen invasion in most Lyme disease patients are characterized by chronic erythema migrans. Several weeks after the disease onset, some patients may develop neurological and cardiac problems. After several months, most patients will have recurrent symptoms such as joint pain or arthritis (63). Under the stimulation by borbora burgdorferi, an accumulation can be detected within the inflated joints of the patients (64), suggesting a possible role for these cells in the host immune responses to the pathogen. Similarly, proliferated γδT cells have also been detected in leukocytes from micewith Lyme disease and in human peripheral blood after tick bites (65). However, the responses of γδT cells may be indirectly through the actions of Toll-like receptors of DCs or monocytes, rather than through a direct mechanism by themselves (64). In addition, γδT cells may also act to activate the host acquired immunity during the infection of the pathogen (66).

#### Tularaemia

The pathogen for tularaemia is Francisella tularensis, a type of gram-negative bacterium, which can be transmitted by tick bites from different hosts and cause acute febrile disease (67). Increased numbers of γδT cells can be detected in the blood from tularaemia patients (67), possibly attributable to non-specific phosphor-molecules (68). Further investigations by M. KROCA and colleagues have revealed that the frequency of γδT cells can be increased and maintained for up tyo a year in the peripheral blood from tularaemia patients (69).

### Immune responses of the host γδT cells to other vector-borne diseases

#### Leishmaniasis

Leishmaniasis is disease caused by the infection of leishmania and primarily transmitted by the bites from different hosts by leishmania-infected female diptera insect phlebotomus fly. Leishmaniasis can be categorized into three major subtypes, including visceral leishmaniasis, cutaneous leishmaniasis, and mucocutaneous leishmaniasis. Visceral leishmaniasis is also called kala-azar (black sickness) and is the most severe subtype of leishmaniasis. Visceral leishmaniasis leads to symptoms including irregular fever, weight loss, hepatosplenomegaly, and anamenia, and may eventually cause patient death. Cutaneous leishmaniasis is the most popular subtype and mainly causes skin ulcer. Mucocutaneous leishmaniasis mainly causes mucous membrane injury within the oral and nasal cavity. Leishmania belongs to parasites, and cellular immunity plays a central role in the host responses to its infection. An accumulation of γδT cells has been detected in the skin and blood from human hosts infected with leishmania (70, 71), suggesting a potential role for γδT cells in eliminating the infected parasites. Consistently, it has been reported that natural killer cells and γδT cells act through secreting INF-γ and TNF-α, respectively, to exert their functions in the host innate immunity against the leishmania invasion (72). Compared with healthy individuals, double negative T cells from about 75% of the cutaneous leishmaniasis patients express aβT cell receptors, and the rest of the double negative T cells express γδT cell receptors (73). In addition, dogs severely infected with leishmania may develop γδT cell lyphoma (71), suggesting that long-term stimulation by leishmania may lead to malignant transformation and lyphoma pathogenesis, but the underlying mechanism has been unclear.

#### South American trypanosomiasis

South American trypanosomiasis is also known as Chagas’ disease and mainly caused by direct contact with the excrement or urinate of Trypanosoma cruzi-infected trypanosoma triatoroae (stink bug). Currently there is no vaccine available for this disease. During the acute stage, the infected patients mainly exhibit symptoms including cyanosis swelling on one side of the skin or eyelid, headache, difficulty in breath, and muscle pains. At the chronic stage, the parasites parasitize in the intestine tracts and the heart. Years later after the infection, some patients may develop heart and digestive tract diseases. By utilizing γδT cell-deficient mice as a model, a recent report has shown that the γδT cells may not play a critical role in the elimination of the parasites at the acute stage of the disease but may contribute to tissue damage and pathogenesis. In cutaneous leishmaniasis patients, αβT cells and γδT cells secret inflammatory factors and IL-10, respectively to protect the hosts against the parasites invasion (74). Moreover, the frequency of IL-10 expression by γδT cells have been linked to an improvement of cardiac functions of cutaneous leishmaniasis patients, suggesting a potentially important role for γδT cells in the host responses (74).

## Conclusions

It is estimated that vector-borne diseases lead to more than half million of global deaths each year, and some types of vector-borne diseases, such as chikungunya, leishmaniasis, and lymphatic filariasis may cause life-long diseases. Vaccines or other clinically effective drugs for many vector-borne diseases are still lacking, further worsening the life quality of the infected patients. As such, understanding better the host-pathogen interactions is critical for future developing novel and curative therapeutics.

Compared to unconventional γδT cells, the role for conventional αβT cells in the host responses to vector-borne pathogens has been more extensively and comprehensively studied, for instance Chikungunya virus (81–83), Rift Valley Fever virus (28), Yellow fever virus (84, 85), Zika virus (86, 87), West Nile virus (88), Malaria (89, 90), Borrelia burgdorferi (91), and Leishmaniasis (73, 92). However, as above-reviewed, our understanding of the role for γδT cells in these processes has been preliminary and incomplete.

It should be noted that most types of pathogens for vector-borne diseases are carried and disseminated by mosquitos and ticks, which transmitted the pathogens through the bites of host skin (93, 94). Given that γδT cells primarily reside in skin and mucosal tissues (95), these cells apparently are in the frontline to respond to pathogen invasion at the earliest stage. Therefore, it is important and urgent to gain a better understanding of the role for γδT cells during these precesses.

Previous studies have shown that αβT and γδT cells cooperate with each other and act synergistically towards eliminating pathogen invasion. As a bridge between innate and adaptive immunity, γδT cells have been known to play active roles during the first and secondary infections by pathogens and may serve as targets for clinical development. However, we still have limited knowledge about details of how they appropriately respond to vector-borne pathogen infection to facilitate the host immune responses.

During the early events of pathogen infection, activated γδT cells can secret multiple cytokines and inflammatory factors to induce the acquired immunity (96–98). Further studies in rodent models of infection of listeria, cytomegalovirus, and plasmodium parasites have revealed that γδT cells can strongly respond and quickly expand during secondary infections, suggesting that they have acquired certain levels of immune memory. These findings also suggest that the mode by which γδT cells respond to pathogen infections may be more complicated than previously appreciated.

Herein, we have reviewed recent findings related to the potential roles of γδT cells in response to several types of vector-borne pathogens, especially the mosquito- and tick-borne pathogens. We expect that these findings, together with those from more studies to analyze the interactions between γδT cells and vector-borne pathogens in the future, will provide useful information for developing clinically relevant targeted therapeutics.

## Author contributions

CC and YY wrote the manuscript; AC and YY contributed to figures in this manuscript. All authors listed have made a substantial, direct, and intellectual contribution to the work and approved it for publication.

## Acknowledgments

This work was partly supported by a junior faculty start-up fund from Anhui Medical University (to CC). We apologize for not being able to cite all important literatures in the field due to space limit.

## Conflict of interest

The authors declare that the research was conducted in the absence of any commercial or financial relationships that could be construed as a potential conflict of interest.

## Publisher’s note

All claims expressed in this article are solely those of the authors and do not necessarily represent those of their affiliated organizations, or those of the publisher, the editors and the reviewers. Any product that may be evaluated in this article, or claim that may be made by its manufacturer, is not guaranteed or endorsed by the publisher.
